# Unveiling novel targets of paclitaxel resistance by single molecule long-read RNA sequencing in breast cancer

**DOI:** 10.1038/s41598-019-42184-z

**Published:** 2019-04-15

**Authors:** Bi Lian, Xin Hu, Zhi-ming Shao

**Affiliations:** 1 Key Laboratory of Breast Cancer in Shanghai, Department of Breast Surgery, Fudan University Shanghai Cancer Center, 270 Dong’an Road, Shanghai, 200032 China; 2 Department of Oncology, Shanghai Medical College, Fudan University, 138 Yixueyuan Rd, Shanghai, 200032 China

## Abstract

RNA sequencing has become one of the most common technology to study transcriptomes in cancer, whereas its length limits its application on alternative splicing (AS) events and novel isoforms. Firstly, we applied single molecule long-read RNA sequencing (Iso-seq) and de novo assembly with short-read RNA sequencing (RNA-seq) in both wild type (231-WT) and paclitaxel resistant type (231-PTX) of human breast cancer cell MDA-MBA-231. The two sequencing technology provide both the accurate transcript sequences and the deep transcript coverage. Then we combined shor-read and long-read RNA-seq to analyze alternative events and novel isoforms. Last but not the least, we selected BAK1 as our candidate target to verify our analysis. Our results implied that improved characterization of cancer genomic function may require the application of the single molecule long-read RNA sequencing to get the deeper and more precise view to transcriptional level. Our results imply that improved characterization of cancer genomic function may require the application of the single molecule long-read RNA sequencing to get the deeper and more precise view to transcriptional level.

## Introduction

With the advancing technology of RNA sequencing (RNA-seq)^1^, the mRNA level of variation can be clearly elaborated by known genes and their different transcriptions, which the number of transcription is 4–5 times than the genes^2^. The complexity of transcriptions and proteins are mainly due to the numerous alternative splicing (AS) events, which is a common phenomenon in eukaryotic species^3^. In human, 95% genes are multi-alternatively spliced, whereas the function of every event is yet to be studied^4^. In terms of cancer research, previous reviews have shown that about 15,000 AS events are detected in 27 cancer types and the cancer specific AS events are correlated to differentiated cancer survival^5^. However, with the development of cancer processing, various number of unannotated AS events happened and formed novel transcription, which may play a critical role in cancer metastasis and drug resistance^6^.

Other than short-read RNA sequencing which assembles short fragments (36–125 bp) and aligns to the reference such as GRCh38, the single molecule long-read RNA sequencing, which also called Pacbio sequencing (Iso-seq) can detect as long as thousands of base pairs (1–10 kb) and assemble as de novo transcriptome reference^7^. Therefore, Iso-seq is widely used in constructing genome reference of different species, which further more expending its application into high-throughput sequencing with de novo genome reference when combining with RNA-seq.^8^.

The chemotherapy resistance has been a challenging project in breast cancer, especially in triple negative breast cancer (TNBC)^9–11^. In spite of the emerging therapeutic such as immunotherapy, the chemotherapy is still the only recognized standard therapy for TNBC patients^12,13^. Thus, the potential drivers and mechanism for drug resistance is necessary to be clarified. Recently, increasing researches have remarkable founding on the function of some alternative splicing event and transcripts played a pivotal role the tumorigenesis, progression, and metastasis of cancer, as well as inducing drug resistance^14–16^.

Here we treated one of the TNBC cell lines, MDA-MB-231 with paclitaxel to generate paclitaxel-resistant cells. Then we used both RNA sequencing and Pacbio sequencing technology to perform paclitaxel resistant cells and wide type cells. We combined two kinds of data to build a paclitaxel-resistant genomic profiling of TNBC. Next, we analyzed the AS events using Iso-seq data and verified with RNA-seq splicing junction. Moreover, we defined 390 differentiated isoforms combing both the RNA sequencing and Pacbio sequencing results and highlighted 11 novel differentiated isoforms with the common character that they can only be detected on isoforms level. At last, we verified a novel isoform of BAK1 genes.

## Methods

### Cell lines and cell culture

The MDA-MB-23 human triple negative breast cancer cell line from the Shanghai Cell Bank Type Culture Collection Committee (CBTCCC, Shanghai, China) and cultured in complete growth medium as recommended by the distributor. The MDA-MB-231 paclitaxel-resistant subline 231-PTX was established by concentrations of paclitaxel (MCE) 1 mM with 12 cycles in culture medium. Each cycle lasts for 24 hours and change for normal culture medium to culture for 2 weeks.

### Cytotoxicity and cell proliferation assays

The Cell Counting Kit-8 (CCK-8) assay (Dojindo Laboratories, Kumamoto, Japan) was used to construct a dose-response curve to obtain half the maximal inhibitory concentration (IC50) of each compound. The IC50 was calculated using the XLFit curve fitting software (Microsoft Inc., CA). All experiments were performed three times in triplicate.

### RNA Extraction, Library Preparation and Sequencing

Total RNA was extracted using TRIzol reagent (Invitrogen) and extracted following the manufacturer’s protocol. For RNA sequencing, the libraries were constructed using TruSeq Stranded mRNA LTSample Prep Kit (Illumina, San Diego, CA, USA). And were sequenced on the Illumina sequencing platform (HiSeqTM 2500). For Pacbio sequencing, cDNA was synthesized using a SMARTer PCR cDNA Synthesis Kit (Clontech) and SMRT bell libraries were generated using a SMRTbell Template prep kit 1.0 (Pacific).

### Iso-Seq sequence processing

The IsoSeq software ver2.3.0 (PacBio) was applied to get the reads of interest (ROI) fragmets and Quiver software was applied for Error Correction (ICE) to generate the cluster consensus sequences. Next, the package Long-Read De Bruijn Graph Error Correction (LoRDEC)^17^ was used to correct the low quality data using RNA sequencing reads of the same experiment^18^. The corrected sequences were mapped to the NCBI Refseq genome by software GMAP^19^. Moreover, the corrected isoforms were collapsed by the ToFU package (http://github.com/PacificBiosciences/cDNA_primer/) with the index of identity = 85% to detruncate the redundant isoforms. Isoforms differing only at the 50 bp of the starting site were defined as redundant isoforms and only the longest version was retained.

### RNA-seq data processing

Raw data (raw reads) were processed using NGS QC Toolkit^20^. Then the clean reads were mapped to reference genome using hisat2^21^. FPKM^22^ fragments Per kb per Million reads) value of each gene was calculated using cufflinks^23^, and the read counts of each gene were obtained by HTseq-count^24^. DEGs were identified using the DESeq package (https://github.com/olgabot/rna-seq-diff-exprn) functions estimate size factors and negative binomial distribution test. P value < 0.05 and fold change >2 or fold change <0.5 was set as the threshold for differential genes. For short-read assembly, short contigs were reassembled by Cufflinks to get the de novo assembly.

### Alternative splicing event analysis

After data processing, PacBio sequencing isoforms were analyzed by the software AStalavista^25^ to detect 5 types of AS events including A3SS, A5SS, ES, IR and MXE. The novel AS event was defined by MatchAnnot package (https://github.com/TomSkelly/MatchAnnot). For RNA sequencing splicing junction investigation, short reads were aligned to the Pacbio sequencing isoforms by STAR^26^ to detect four splice motifs including CT/AC, CT/GC, GT/AT and other non-canonical sites.

### Novel isoform analysis

The PacBio isoforms were mapped to reference genome using MatchAnnot. The novel transcripts were defined as the coverage <50% of the annotated isoforms. The novel transcripts identification in RNA sequencing were the result of comparing de novo assembly to the reference genome using cuff compare software. Iso-seq novel isoforms were aligned with RNA-seq novel isoforms by BLAST+ package (http://blast.ncbi.nlm.nih.gov) with the index of coverage > = 0.85, identity > = 90 and e value < = 1e-20 to obtain the intersection of the two datasets. To create Unigene, a new genome reference, the novel isoforms to were compared to the NCBI Refseq by bowtie2 (http://bowtie-bio.sourceforge.net/bowtie2/manual.shtml) and eXpress (http://www.rna-seqblog.com/express-a-tool-for-quantification-of-rna-seq-data/) to acquire abundance of Unigene transcripts of the two samples. Then FPKM was applied for quantifying the Unigene expression. The fold change values and *p* value were calculated by DESeq, with threshold pf fold change >2 or <0.5 and *p* value < 0.05. We applied the Integrated Genomics Viewer v2.3.55 to visualize the region of BAK1^27^.

### Real-time PCR and PCR

Real-time PCR was performed to detect selected transcripts using a SYBR Premix Ex Taq System (TaKaRa). PCR was performed to validate the novel isoforms by Q5 High-Fidelity DNA Polymerase (NEB). Primers are listed in the Supplementary Table.

## Results

### Data profile of MDA-MB-231 parental and resistant cells

**Figure 1:**
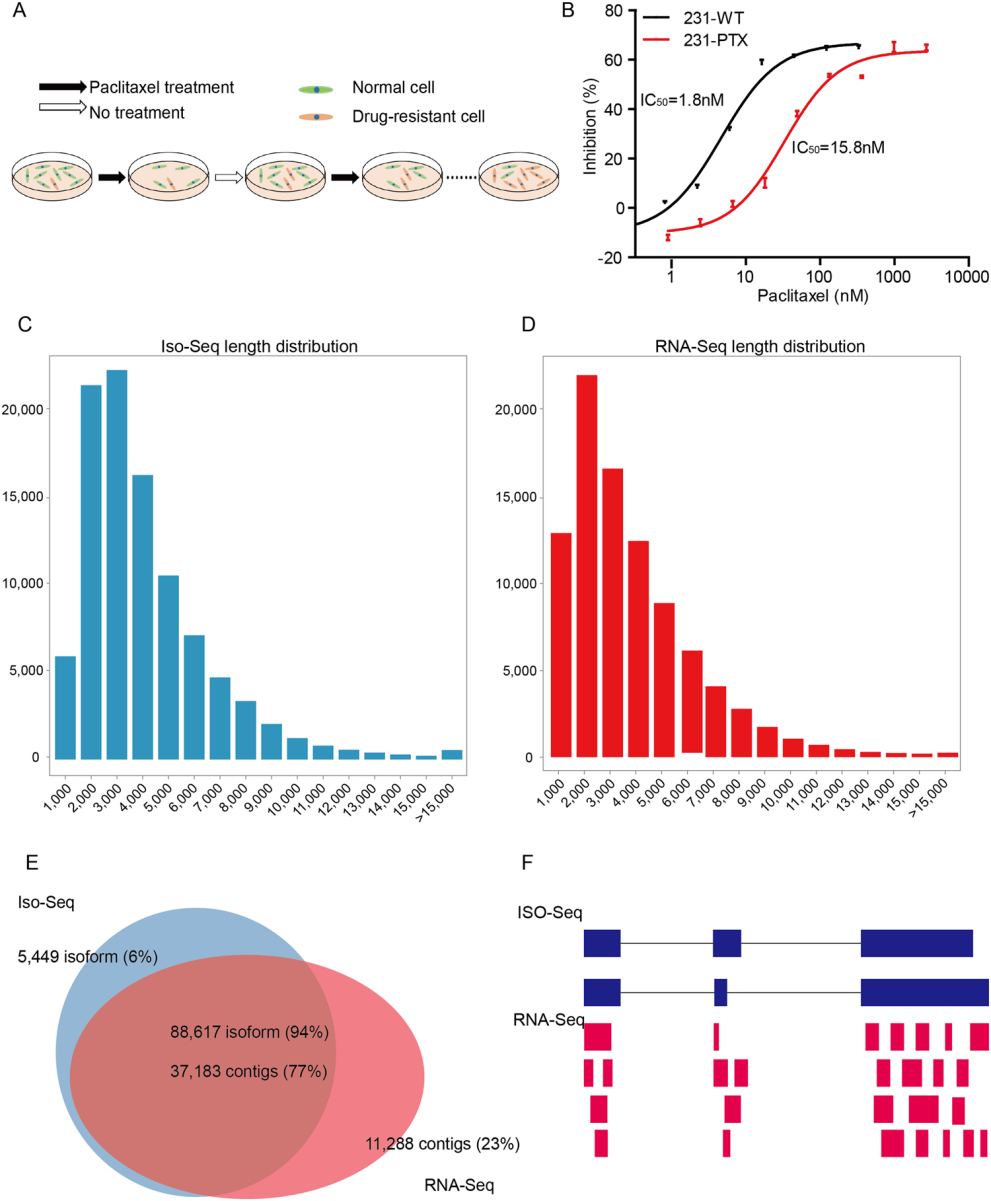
Paclitaxel resistant subline profile and comparison between Iso-seq and RNA-seq. (**A**) Paclitaxel resistant subline 231-PTX was established after 12 cycles of exposure to high concentration of paclitaxel. (**B**) 231-PTX and 231-WT cells were treated with different doses of paclitaxel. IC50 values of the two samples were determined by CCK8 assays, which were performed in triplicate. (**C**,**D**) The length distributing of Iso-seq and RNA-seq displayed in histogram. (**E**) The venn diagram displayed the consistent rate of Iso-seq isoforms and RNA-seq assembling contigs. (**F**) The diagram visualized the different view of Iso-seq and RNA-seq to analyze transcripts.

In order to construct a paclitaxel-resistant cell subline of MDA-MB-231 (231-PTX), we treated cells as described in Materials and Methods (Fig. 1A). The paclitaxel half-maximal inhibitory concentration (IC50) value for 231-PTX cells was 10.7 nM, which was nearly ten fold higher than that for the 231-WT cells (1.63 nM) (Fig. 1B). After establishing the stable drug resistant cell line, the 231-PTX and 231-WT were performed by RNA-seq and Iso-seq.

To avoid the bias of preferentially detecting shorter length of isoforms, three SMRT Cells with the read length of <2 kb, 2–3 kb and >3 kb were sequenced and produced about 5000 million bases data for each cDNA sample. The base pair quality, read length and coverage of Reads-Of-Insert (ROI) of the two samples indicated that the Iso-seq data was in high quality, wide range length and adequate ROI coverage (Supplementary Fig. 1A–F). Following the standard TAPIS workflow, the full-length non-chimeric ROI reads and non-full-length ROI reads, which were retained for further analysis were 115,319 and 84,401 for 231-WT and 101,126 and 70,485 for 231-PTX (Table 1). After correction by RNA sequencing fragments and removing the truncated isoforms, the total numbers of isoforms are 26,268 for 231-WT and 23,784 for 231-PTX (Table 1).

**Table 1:** Statistics profile of PacBio Iso-seq and Illumina RNA-seq in 231-WT and 231-PTX.

Sample	MDA-MB-231-WT	MDA-MB-231-PTX
**PacBio Iso-seq**		
Polymerase Reads		
Reads	238,068	203,546
Base	5,905,208,385	4,834,545,014
Mean Length	24,804	23,751
Sub-reads		
Reads	3,227,804	2,707,467
Mean Length	1,787	1,744
ROI reads		
Reads	214,870	187,371
Full-length non-chimeric reads		
Reads	115,319	101,126
Mean Length	2,087	2,054
Non-full-length reads		
Reads	84,401	70,485
Isoforms after correction		
Reads	49,189	45,083
Mean Length	2,374	2,239
Isoforms after detruncating		
Reads	26,268	23,784
MeanLength	2,375	2,283
**RNA-Seq**		
Reads		
Total Reads	1: 47,075,474 2: 47,214,460	1: 47,141,024 2: 46,163,182
Uniquely mapped	1: 41,427,032(88.00%) 2: 41,519,944(87.94%)	1: 42,226,893(89.58%) 2: 41,514,133(89.93%)
Junctions		
Number	1: 241,744 2: 243,052	1: 251,480 2: 249,983

In terms of RNA-seq results, two replicates of each sample were sequenced to obtain about 47 million clean reads. The number of genes that covered 90–100% of the whole length by RNA-seq fragments were mapped nearly 77% of GRCh38 gene symbols (Table 1). The reads distribution of every samples and the correlation between replicates and samples were displayed in Supplementary Fig. 2A,B.

### De novo assembly of RNA-seq contigs

The RNA-seq clean reads were assembled by Cufflinks to obtain RNA-seq contigs. The number of Iso-seq isoforms were retained its highest level at 3,000–4000 bp interval, with 1000 longer than that of RNA-seq contigs (Fig. 1C,D). This demonstrated that Iso-seq had advantages covering large transcripts than RNA-seq.

When compared to Iso-seq transcript isoforms, 77% (37,183) of the RNA-seq contigs mapped to 94% (88,617) of the PacBio transcript isoforms. There were 23% (11,288) of RNA-seq contigs and 6% (5,449) of Iso-seq isoforms unique to each of the datasets due to the fake assembly of RNA-seq short reads and the failed coverage of Iso-seq isoforms to all the human genome sequences (Fig. 1E). Consequently, the Iso-seq long-length isoforms provide a precise and comprehensive perspective of the whole transcriptome and the RNA-seq short-length fragments give the information of the count of each transcript (Fig. 1F).

### Novel alternative splicing events predominate in 231-WT and 231-PTX

**Figure 2:**
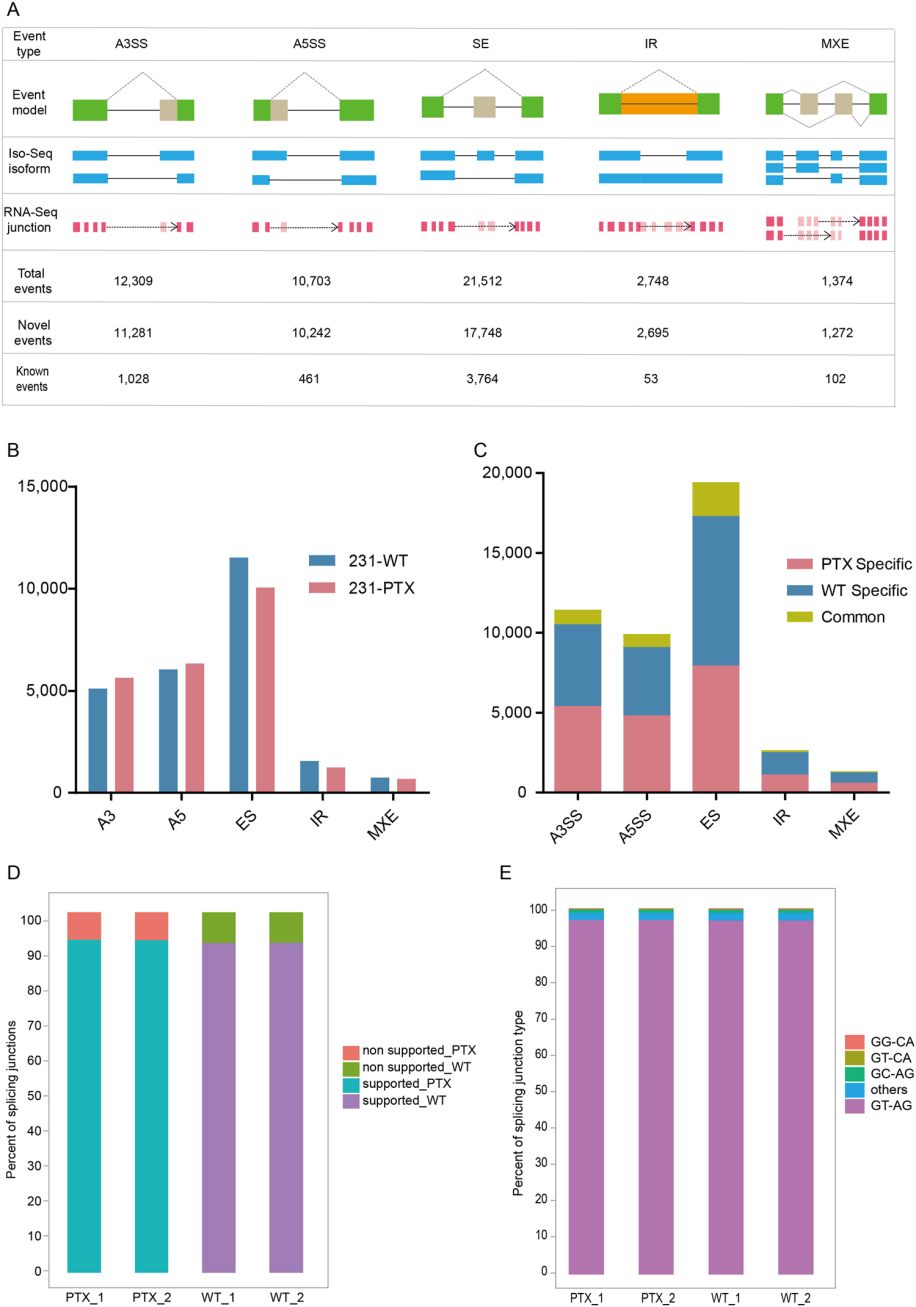
Dramatic changes of AS events in 231-WT and 231-PTX. (**A**) Diagrams displayed the five alternative RNA processing events and the event number of each type. AS events include 5 types, as shown in the figure, that are skipped exons (SE), alternative 5′or 3′ splicing sites (A5SS or A3SS), internal retention (IR) and mutually exclusive exon (MXE). (**B**) The constitution of each AS type in the two samples of 231-PTX and 231-WT displayed in histogram. (**C**) The ratio of 231-WT and 231-PTX specific and common AS event of each type displayed in histogram. (**D**) The splicing junction matching the AS event in Iso-seq of each sample displayed in histogram. (**E**) The constitution of splicing junction model of each sample displayed in histogram.

Based on Iso-seq, we identified the number of each AS event type in 231-WT and 231-PTX (see Materials and Methods). In total, we detected 48,646 AS events in Iso-seq isoforms of both 231-WT and 231-PTX samples. Among these, SE event was the most prevalent mode, which was consistent with previous research. Compared to annotated AS events in Refseq, only 11.11% of all the AS events were mapped, which illustrated that a vast viability of AS events during paclitaxel resistance process in TNBC (Fig. 2A). The constitution of AS mode of the two sample were coincident, which indicated the AS mode was not changing during the process of turning into paclitaxel resistant cells (Fig. 2B). However, we discovered that over 90% of the events were specific to one sample, which illustrated the AS event of each AS type changed dramatically during that process (Fig. 2C).

Since the RNA-seq defining AS events at a local view, we used it to evaluate the accuracy of Iso-seq results. An average of more than 90% of the Iso-seq junction positions were supported by RNA-seq short reads, which indicating high accuracy of the Iso-seq in analyzing the alternative splicing events (Fig. 2D). Another alignment method was to detect the detailed base-pair alteration of splicing junctions. It was suggested that GT/AG consists more than 95% of all the junctions were all the supported junctions (Fig. 2E).

### Differentiated novel isoforms outstand in inducing drug resistance

**Figure 3:**
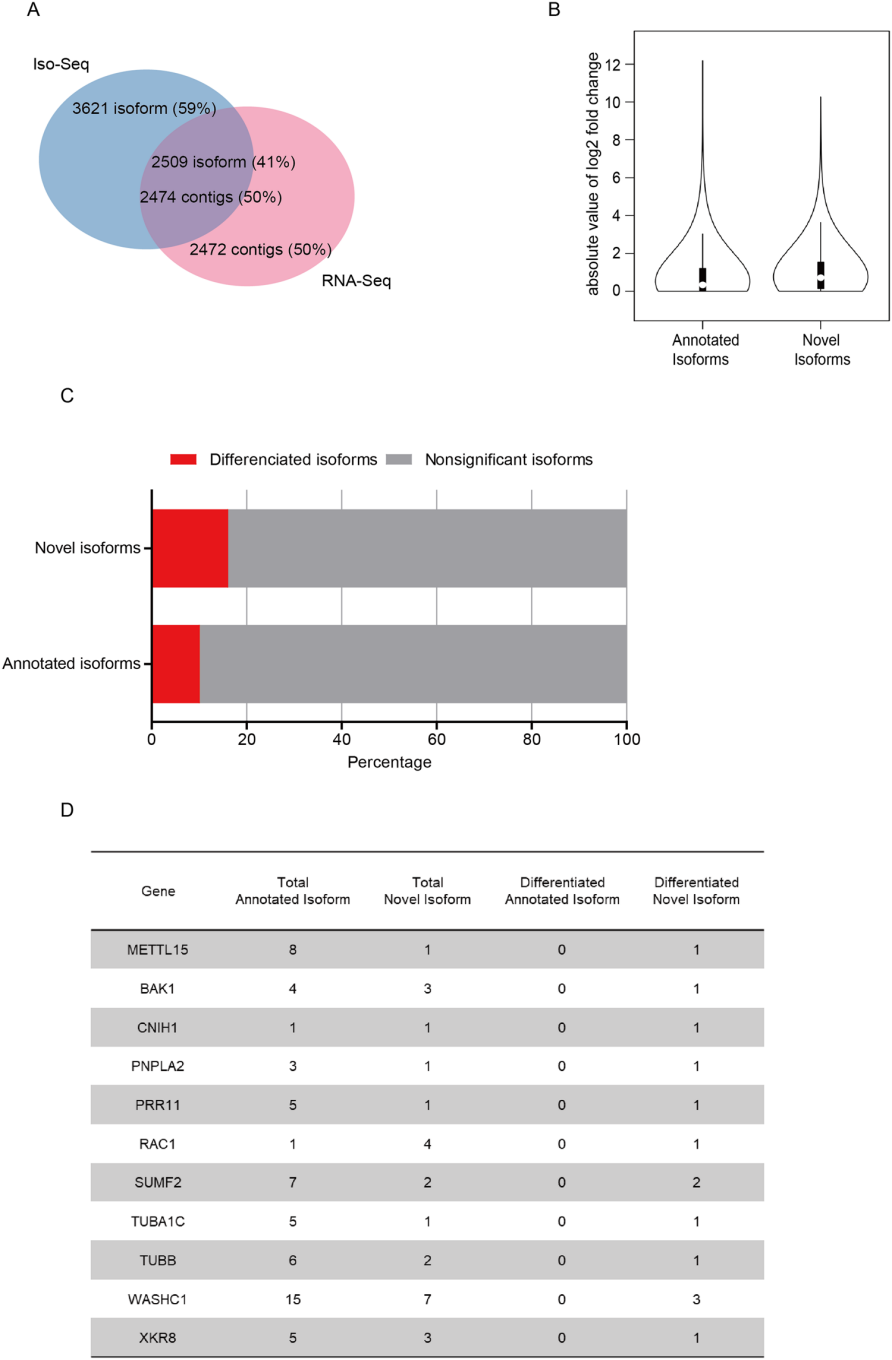
Potential transcriptional biomarkers related to drug resistance in TNBC cell. (**A**) Novel isoforms interaction of Iso-seq and RNA-seq results displayed in pie chart. (**B**) The violin plot demonstrated the absolute value of log2 fold change distribution of annotated isoforms and novel isoforms. (**C**) The column bars displayed the percent of differentiated expressed isoforms in novel and annotated isoforms. (**D**) The gene list was selected genes with differentiated novel isoforms but not differentiated annotated isoforms.

Since the majority of the alternative splicing events happened during the process of drug resistance were novel, there should be numerous novel isoforms emerged, whose significance are unexplored. We mapped 79,181 Iso-seq isoforms to NCBI RefSeq database, it turned out 72,602 (91.69%) isoforms in Iso-seq could be annotated and number of novel isoform was 6130 (15.07%). Next we aligned the 6,130 Iso-seq, 4,948 RNA-seq novel contigs, which was displayed in the pie chart that 2,509 Iso-seq isoforms, and 2,474 RNA-seq contigs were matched (Fig. 3A). Therefore, the match isoforms were set as the novel isoforms set for further research.

As the isoforms detected in Iso-seq were not fully covered the Refseq, we combined the Iso-seq isoforms and NCBI Refseq database to create a database called Unigene. The RNA short reads were mapped to Unigene for quantitative-differentiated analysis. The violin plot exhibited that the average value of log2 fold change was higher in novel isoforms than in annotated isoforms (Fig. 3B). In addition, the histogram suggested that there were more proportion of differentiated isoforms in novel isoforms than in annotated isoforms (Fig. 3C). Consequently, the novel isoforms set had more intense alterations comparing 231-PTX to 231-WT. Furthermore, we detected all the genes, which had differentiated novel isoforms but not differentiated annotated isoforms. There was only one gene, METTL15, which also had differentiation on genetic level. However, the rest genes cannot be discovered differentiation on genomic level, which suggested that the precise analysis on transcripts were necessary on these genes (Fig. 3D).

### Verification work of BAK1

**Figure 4:**
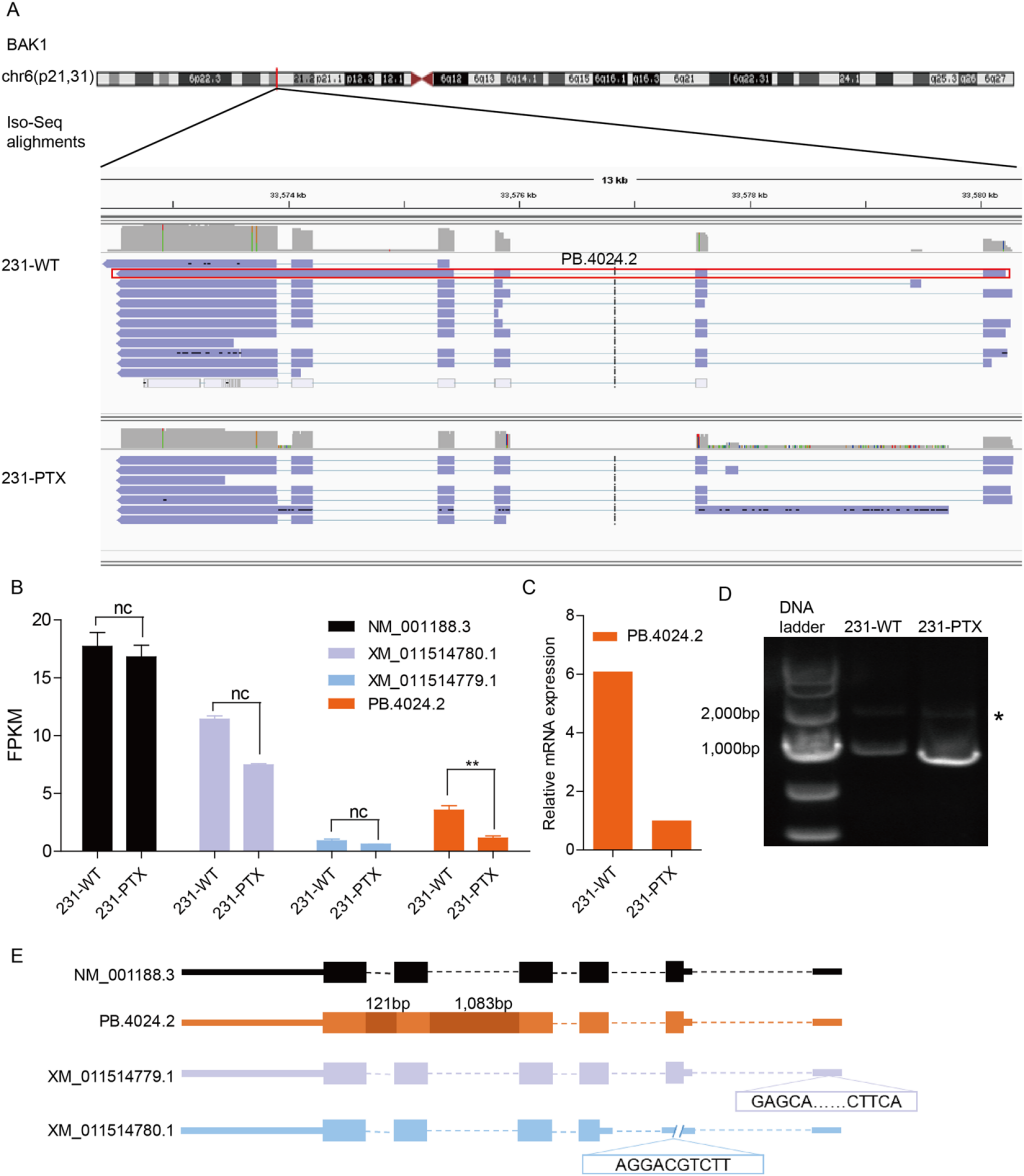
The potential biomarker of PB.4024.4 in paclitaxel sensitivity. (**A**) IGV viewed the isoforms detected in Iso-seq in 231-WT and 231-PTX samples. (**B**) The RNA-seq FPKM value of the four transcripts in BAK1 displayed in histogram.(**C**) Agarose electrophoresis showed that there were two bands emerged in both 231-WT and 231-PTX samples. Novel isoforms were shown with dot mark. (**E**) The model of BAK1transcripts displayed the detailed difference among the isoforms.

From the 11genes listed in Fig. 3E, we chose BAK1 as our verification target. BAK1, as short for BCL2 antagonist/killer 1, is a member of BCL2 family and play roles in inducing apoptosis. If only considered gene expression, BAK1 was not a differentiated gene comping 231-PTX and 231-WT (Supplementary Fig. 3A,B). According to Iso-seq analysis, BAK1 had one novel transcript, PB.4024.2, which was the result of alternative splicing between exon 4 to 5 and5 to 6 (Fig. 4A). When it turned to the transcriptional level, PB.4024.2, was decreased more than three times comparing 231-PTX to 231-WT, while the other three annotated isoforms had no significant change (Fig. 4B). Further verification work including qPCR and PCR experiments turned out to be positive results, which suggested that PB.4024.2 might be the next biomarker in paclitaxel therapy (Fig. 4C,D). The model of all the BAK1 transcripts implied that the AS event of intron retention might be the reason to cause the differentiation among isoforms (Fig. 4E).

## Discussion

Numerous researches have performed sequencing on genetic level to analyze genome profile of drug resistance. Some contribution has been achieved on alternative splicing events of some gene and drug resistance in TNBC. Liu *et al*. applied microarray technology to find the significance of AS events of TRA2A in paclitaxel resistant TNBC cells^28^. Johnson *et al*. induced high lung-metastatic TNBC cell lines and performed RNA sequencing to identify a common dysregulated splicing event in CPEB2^29^. However, a comprehensive analysis of AS event and novel isoforms in TNBC drug resistance has not been reported yet. In this research, we applied the MDA-MB-231 paclitaxel resistant cell line to analyze their AS event character and discovered novel isoforms that were differentiated expressed in 231-PTX compared to 231-WT, which established the basis of the whole-transcriptional expression profile and shed light on the drug resistant research work to a deeper level.

Although the major analyzing strategy of alternative splicing events is to use the short reads from next generation sequencing, it cannot be denied that it is limited to accurately define these events by short RNA-seq reads alone, especially for the IR events^30^. Yet PacBio sequencing provided a direct and convincing approach to identify alternative RNA processing events, as its long length of reads can be clearly distinguished each AS event even the long intron retention event^31^. Therefore, we applied the Iso-seq technology and substantially expanded the repertoire of AS events in TNBC, which had much more number than known events, especially the IR event, with 40 times more novel event detected than the known ones.

Other than species that need Iso-seq to identify their genome, the human genome has established several comprehensive genome database, among which the most widely used is GENCODE. However, the diversity of novel isoforms in human genome in different situations makes it necessary to use Iso-seq technology to precisely discovered dramatic alterations on isoform level. Shi *et al*. applied SMRT PacBio sequencing on Chinese individual HX1 to explore the Chinese genome profile specifically^32^. Targeted TP53 sequencing PacBio dataset and FLT3 PacBio sequencing dataset were established by Olivier *et al*. and Gilliland *et al*. for clinical use^33,34^. Our research is the first to use the PacBio sequencing on studying drug resistance in breast cancer. We focused on the novel isoforms that were not only significantly expressed but also would be missed if only performing RNA sequencing. The genes listed are worth studying their different functions between isoforms and may be the potential biomarker on drug resistance.

Despite its long length reads, PacBio Iso-seq is currently not good enough in comparative analyses due to its low throughout. While RNA-seq has the high throughout but short length reads^35^. Therefore, we adopted the common novel isoforms of the two sequencing technology to establish a new genome reference called Unigene, which was mapped by RNA-seq short reads to calculate expression and compare the expression of the two samples. We focused on the novel isoforms that were not only significantly expressed but also probably be missed if only performing RNA sequencing. The genes listed are worth studying their different functions between isoforms and may be the potential biomarker on drug resistance.

BAK1 is one of the BCL family members and functioned as a apoptosis inducing gene^35^. Previous studied have depicted that BAK1 played a role in tumor growth and drug sensitivity. Aarnink *et al*. discovered that in 69 gastric cancer patients after chemotherapy whose specimens showed good chemotherapeutic histopathological responses had higher BAK expression than those with poor responses^36^. However, there is few evidence about BAK1 in breast cancer. Our definition of the novel isoform, PB.4024.2 is the potential biomarker of drug resistance in TNBC.

**Figure 5:**
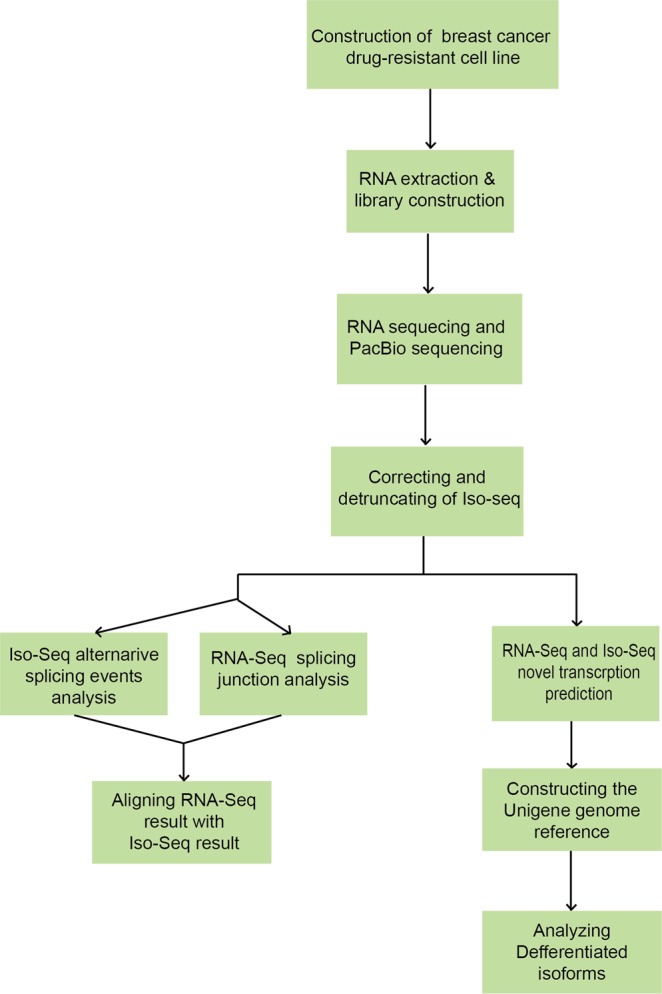
The strategy of analyzing alternative splicing event and novel isoforms from Iso-seq and RNA-seq. The flow chart elaborated our technology from head to toe, including RNA processing, data processing, assembling to mapping the reference and analyzing AS events and novel isoforms in the two samples.

In conclusion, we detected a new strategy to analyze AS events and novel isoforms and pave a new path to drug resistance research in cancer (Fig. 5). Limited by the bio informatics technology, the analyzing results in our research are only a primary step. Deeper analyzing is required for the current results and further research is ongoing for studying the function of PB.4024.2.

## Supplementary information

Supplementary information

## Data Availability

We declare that materials described in the manuscript, including all relevant raw data, will be freely available for non-commercial purposes.
