# Supplementary material for: Unveiling novel targets of paclitaxel resistance by single molecule long-read RNA sequencing in breast cancer
Source: Sci Rep. 2019 Apr 15;9:6032. doi: 10.1038/s41598-019-42184-z (PMC6465246; doi:10.1038/s41598-019-42184-z)

Unveiling novel targets of paclitaxel resistance by single molecule long-read RNA sequencing in breast cancer

Bi Lian[1, 2], Xin Hu[1,2,*], Zhi-ming Shao[1,2,*]

Supplementary information

Supplementary table: real-time PCR and PCR primers

Real-time PCR	
BAK1-F	GTTTTCCGCAGCTACGTTTTT
BAK1-R	GCAGAGGTAAGGTGACCATCTC
PB.4024.2-F	CAGGTAATCTCCCCGCTGAC
PB.4024.2-R	GCTGTGAATTAAGGCCGCAG
PCR	
BAK1-F	GCTGATCCCGTCCTCCACTG
BAK1-R	TGCTAAAGCTTCTGTACTCT

Supplementary figure 1: (A) 231-WT ROI base pair quality (B) 231-PTX ROI base pair quality (C) 231-WT ROI read length (D) 231-PTX ROI read length (E) 231-WT ROI coverage (F) 231-PTX ROI coverage

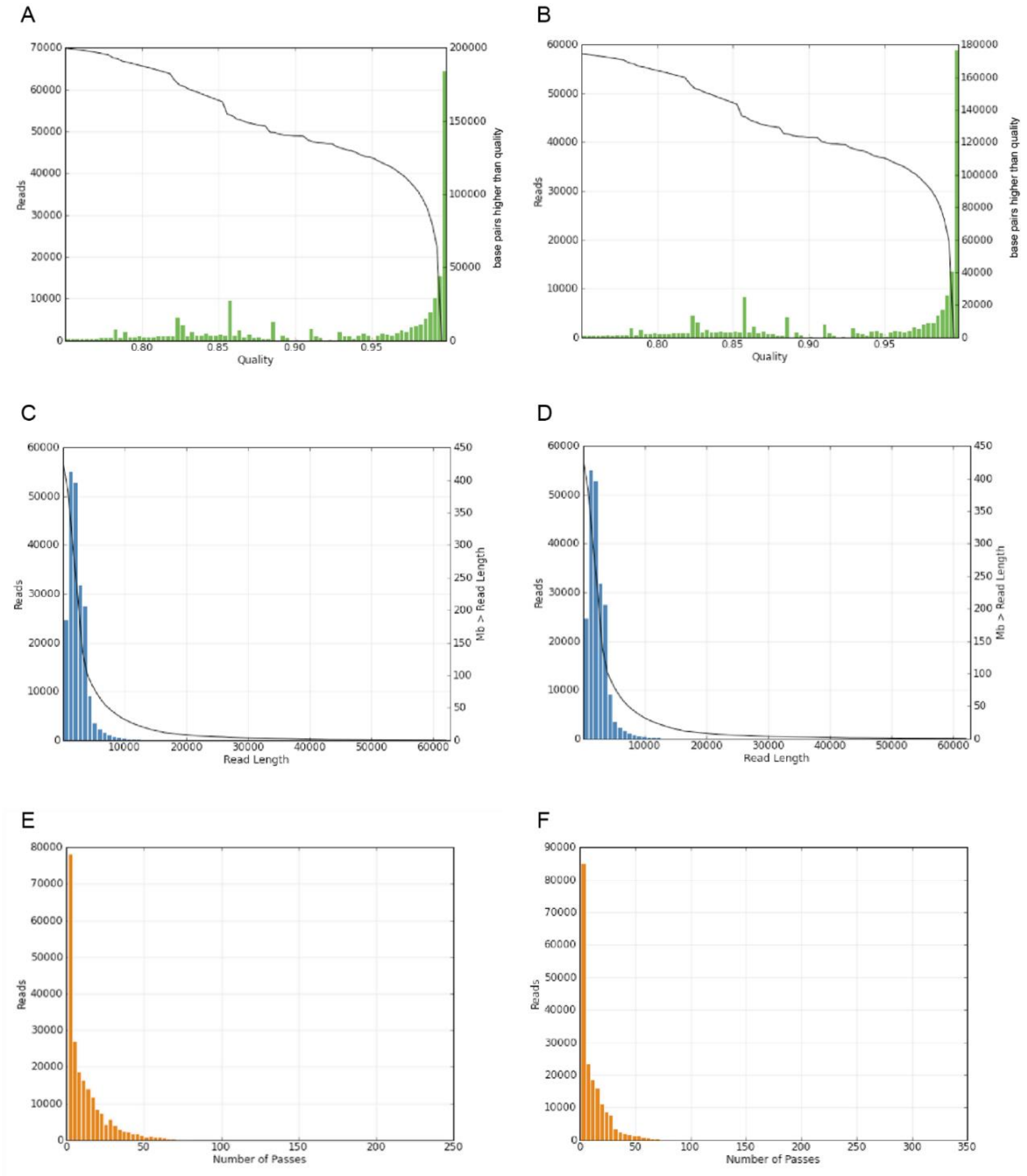

Supplementary figure 2: (A) RNA-seq transcript expression boxplot (B) Correlation among replicates and samples

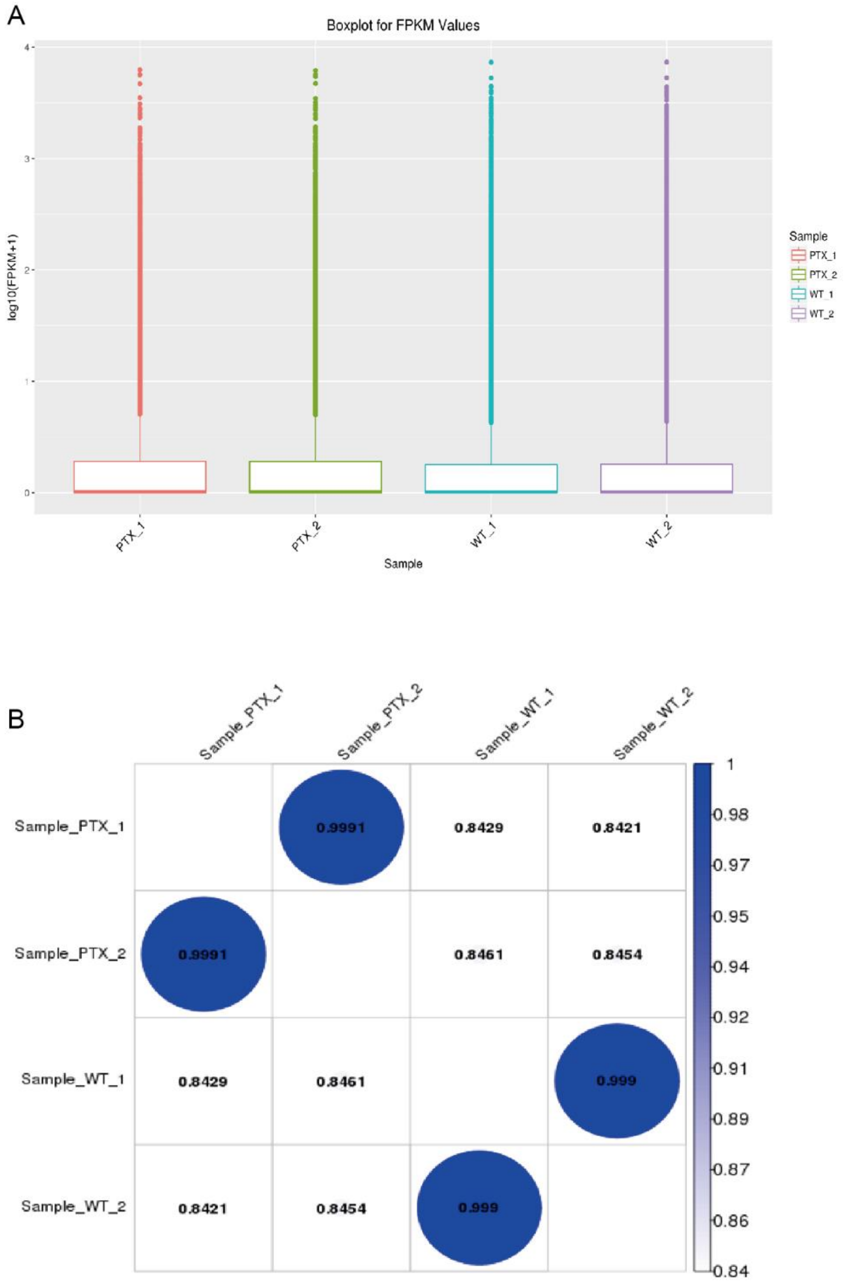

Supplementary figure 3: (A) IGV view of short reads covering the domain of BAK1 (B) The FPKM value of 231-PTX and 231-WT, which indicated that BAK1 had no statistical significance.

A

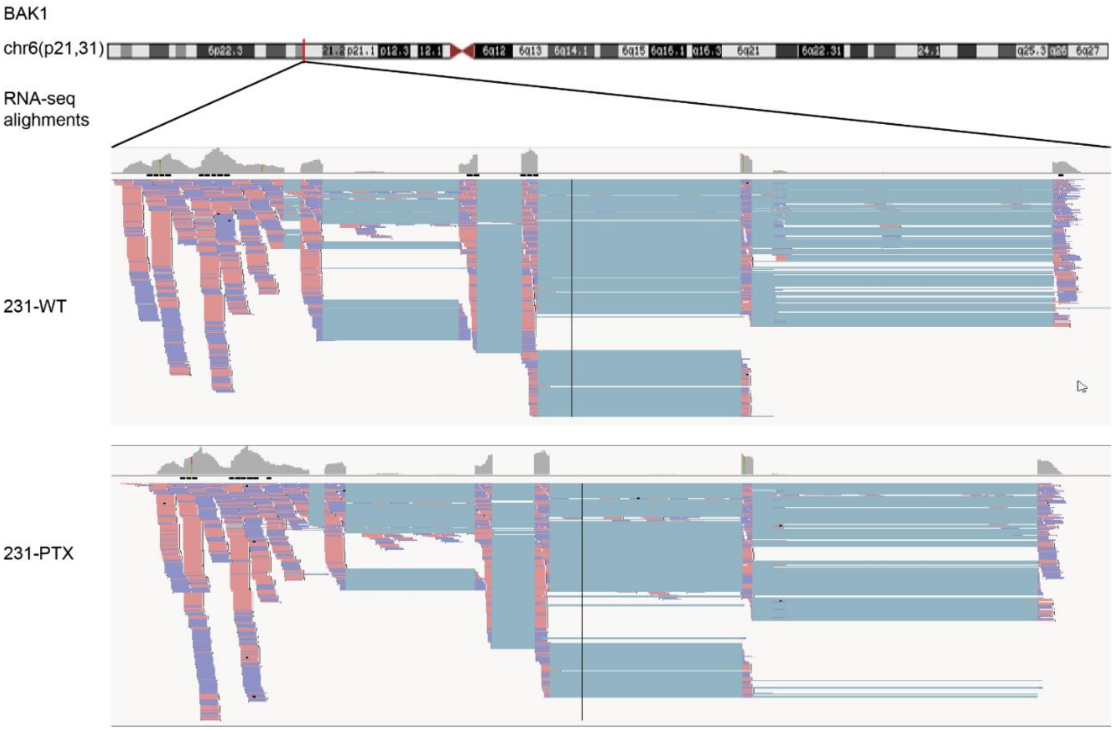

B

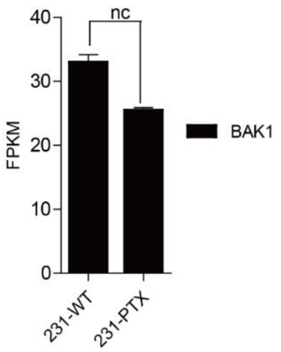

Supplement: Supplementary file 1 — Supplementary information [file 41598_2019_42184_MOESM1_ESM.pdf]
